# New strategy for antimetastatic treatment of lung cancer: a hypothesis based on circulating tumour cells

**DOI:** 10.1186/s12935-022-02782-w

**Published:** 2022-11-15

**Authors:** Zujun Que, Jianhui Tian

**Affiliations:** 1 Institute of Oncology, Shanghai Municipal Hospital of TCM, Shanghai University of Traditional Chinese Medicine (TCM), Shanghai, 200071 People’s Republic of China; 2 Clinical Oncology Center, Shanghai Municipal Hospital of TCM, Shanghai University of TCM, Shanghai, 200071 People’s Republic of China

**Keywords:** Lung cancer, Circulating tumour cells, Antimetastatic, Therapeutic strategy, Biomarker

## Abstract

Metastasis is the primary cause of death in lung cancer patients. However, until now, effective drugs and intervention strategies for treating lung cancer metastasis have been lacking. This hypothesis focuses on circulating tumour cells (CTCs) to develop a new antimetastatic therapeutic strategy for lung cancer. Here, we outline the role of CTCs in tumour metastasis and their functional effects during the treatment of lung cancer patients. Additionally, we hypothesized the possibility of CTCs as a novel biomarker and therapeutic target in preventing and treating metastasis in patients with early-stage lung cancer. We hope that the realization of this hypothesis will improve the overall survival of lung cancer.

## Background

Metastasis is a major factor contributing to the high mortality of lung cancer, and effective antimetastatic drugs are lacking [1]. The primary reason is that the current therapeutic drugs and strategies are based on the molecular or pathological diagnosis results of the primary tumour tissue [2]. However, the benefit for patients with early-stage lung cancer is very limited [3]. Considerable heterogeneity exists between the primary tumour and different metastatic lesions [4]. One tissue biopsy cannot accurately capture the complete genome of the patient’s cancer, and the phenotype and gene of cancer cells will change after treatment, bringing challenges to individualized and precise medication [5]. Additionally, after surgery in patients with early-stage lung cancer, distinguishing between the occurrence and location of metastases is difficult. However, circulating tumour cells (CTCs), as the link between the primary tumour and metastasis, play a crucial role in the precise treatment of lung cancer metastasis [6].

The metastatic cascade of tumours begins before the first diagnosis [7]. Circulating tumour cells (CTCs), shed from primary tumours and circulation in peripheral blood, are considered a precursor of metastasis [8]. CTC counts can be used not only to diagnose lung cancer early but also to assess metastatic risk and prognosis [9, 10]. Multiple lines of evidence have indicated that the metastatic potential of CTCs is significantly enhanced when they form cell clusters with CTCs, neutrophils, and platelets [11], and lung cancer patients with detectable CTC clusters had shorter progression-free survival (PFS) and overall survival (OS) [12]. Additionally, analysing CTCs can reflect not only the primary tumour but also information on undiscovered micrometastasis [13]. Exome sequencing of lung cancer CTCs can identify mutations associated with metastatic cancer [14]. Therefore, analysing ALK, EGFR and other gene mutations and PD-L1 protein expression in CTCs can help clinicians guide medication [15, 16]. Additionally, CTCs can be cultured ex vivo to identify new mutations and perform individualized testing of drug susceptibility [17]. Notably, multiple lines of evidence have indicated that the increased number of CTCs in postoperative patients was consistent with the imaging results of computed tomography (CT) scans and was associated with shorter PFS and OS, suggesting that the existing treatment failed to improve patient survival [18]. Additionally, although no change was found in the number of CTCs, its phenotype changed, leading to treatment resistance and disease progression [19]. In this setting, the formulation of antimetastatic therapeutic strategies based on CTCs may break through the curative effect of lung cancer.

### Hypothesis of anti-metastatic therapy for lung cancer

Anti-metastatic therapy of lung cancer based on CTCs molecular information.

#### Hypothesis-testing

**Fig. 1:**
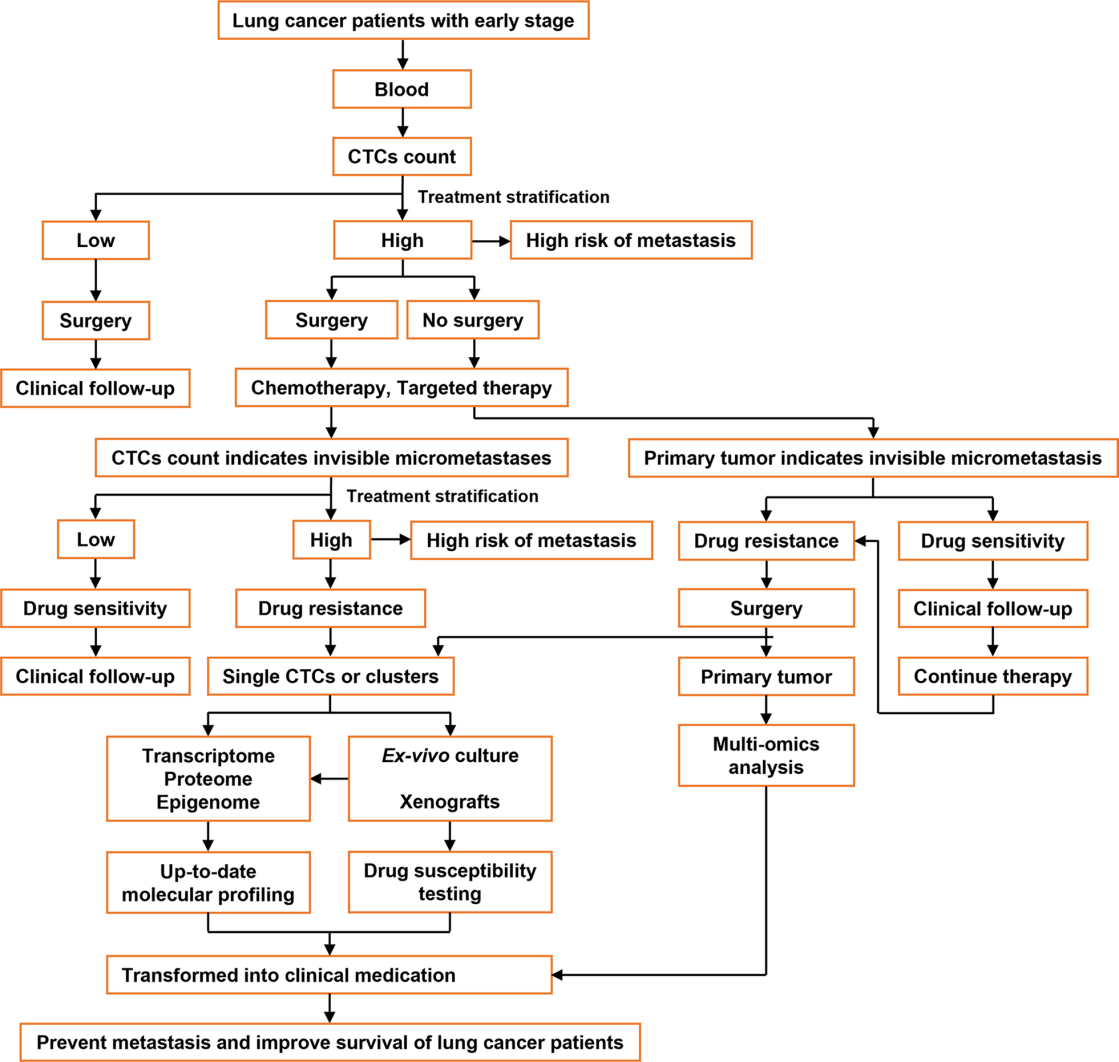
Hypothesis of therapeutic strategies against lung cancer metastasis based on CTCs. Many common mutational gene and protein expression profiles exist among primary tumours, CTCs, and metastases. Primary tumours can reflect invisible micrometastases in response to drug therapy. Additionally, the dynamic change in the CTCs number can also reflect the growth of distant invisible micrometastases, as well as the response of invisible micrometastases, to drug therapy. Targeting CTCs to treat invisible micrometastasis may prevent metastasis and prolong the survival of lung cancer patients

Patients with early-stage lung cancer may already have undetectable micrometastases. Firstly, the risk of metastasis can be stratified by counting CTCs. If CTCs are not detectable in the patient’s peripheral blood or are within a safe threshold range, the risk of metastasis is low, and surgery can be used to prevent metastasis. When a higher number of CTCs are detected in lung cancer patients, it indicates that the patient has a higher risk of metastases or that undetectable micrometastases have occurred. At this time, patients usually undergo surgery and chemotherapy or molecular targeted therapy based on the pathological diagnosis of the primary tumour tissue. Since the clinical efficacy indicators, such as the objective response rate (ORR), are not applicable, the process of evaluating the dynamic changes in CTCs number may be a good alternative index. The analysis of CTCs has the advantages of being real-time, dynamic and repeatable, which can compensate for the lack of tissue biopsy [16]. Secondly, dynamic analysis of CTCs phenotypes or gene mutations, as well as drug susceptibility testing in vitro, can guide the selection of effective therapeutic drugs [20]. The increase in the number of CTCs in patients during treatment indicates that the tumour has progressed and drug resistance has occurred [21]. At this time, CTCs can be analyzed to find new mutant genes and proteins, as well as sensitive therapeutic drugs. These drugs based on the message of CTCs may also have an inhibitory effect on invisible micrometastases (Fig. 1).

Finally, the primary tumour can also be used to indicate the response of invisible micrometastases to antimetastatic therapy. Primary tumours and invisible micrometastases share most of the same mutant genes and protein expression profiles. Drugs that are effective against primary tumours must also be effective against invisible micrometastases. When the primary tumour is resistant to existing therapeutic drugs, then surgery will be performed, and the changes in newly generated mutated genes and protein expression profiles in primary tumours will be analysed to guide clinical drug use. At this time, the therapeutic effect of the drug can still be reflected by analysing the change in the number and phenotype of CTCs. An increase in the number of CTCs indicates new drug resistance. The expression of mutant genes and proteins in CTCs must then be reanalysed to guide clinical medication until metastatic lesions are finally detected by imaging (Fig. 1). Overall, the routine detection and analysis of CTCs can provide clinically relevant information for the timely selection of personalized therapies, possibly leading to improved efficacy of anti-lung cancer metastasis therapy.

## Conclusions

In summary, the primary tumour represents the visible battlefield, and the prevention and treatment of tumour metastasis represent the invisible battlefield. CTCs are associated with distant metastasis and are alternative markers of invisible micrometastases, which, like a beacon in navigation, can dynamically reflect the state of metastases in real time and point out the direction for metastasis prevention and treatment. In the case of tumours in patients with CTCs after surgery, treatment should be adjusted, and multimodal treatment based on the molecular pathological information of CTCs should be considered. CTCs counts are now gradually entering the clinical staging system. Because the analysis and detection standards of CTCs are not unified and the existing clinical treatment guidelines do not allow the primary tumour to guide anti-lung cancer metastasis treatment, clinical trials have not yet been performed. However, lung cancer metastasis may be prevented and the survival of patients may be prolonged. Overall, this hypothesis provides a new strategy for treating lung cancer metastasis and may be of great significance in improving the survival of lung cancer patients.

## Data Availability

Not applicable.

## Abbreviations

CTCs: Circulating tumor cells
CT: Computed tomography
PFS: Progression-free survival
ORR: Objective response rate
OS: Overall survival
